# Humoral and cellular immune responses in mice against secreted and somatic antigens from a C*orynebacterium pseudotuberculosis* attenuated strain: Immune response against a *C. pseudotuberculosis* strain

**DOI:** 10.1186/s12917-016-0811-8

**Published:** 2016-09-08

**Authors:** Vera Lúcia Costa Vale, Marcos da Costa Silva, Andréia Pacheco de Souza, Soraya Castro Trindade, Lília Ferreira de Moura-Costa, Ellen Karla Nobre dos Santos-Lima, Ivana Lucia de Oliveira Nascimento, Hugo Saba Pereira Cardoso, Edson de Jesus Marques, Bruno Jean Adrien Paule, Roberto José Meyer Nascimento

**Affiliations:** 1 Department of Exact and Earth Sciences, State University of Bahia, Campus II, Alagoinhas, BA CEP 48110-100 Brazil; 2 Department of Biointeraction, Federal University of Bahia, Av. Reitor Miguel Calmon s/n, Vale do Canela, Salvador, BA CEP 40110-100 Brazil; 3 Department of Health, Feira de Santana State University, Avenida Transnordestina s/n, Novo Horizonte, Feira de Santana, BA CEP 44036-900 Brazil; 4 Department of Life Sciences, State University of Bahia, Rua Silveira Martins 2555, Cabula, Salvador, BA CEP 41150-000 Brazil; 5 Immunology and Molecular Biology Laboratory, Health Sciences Institute, Federal University of Bahia, Av. Reitor Miguel Calmon s/n, Vale do Canela, Salvador, BA CEP 40110-100 Brazil

**Keywords:** *Corynebacterium pseudotuberculosis*, Cytokines, BALB/c, IgG isotypes

## Abstract

**Background:**

*Corynebacterium pseudotuberculosis* is the etiologic agent of caseous lymphadenitis (CL), a chronic disease that affects goats and sheep. CL is characterized by the formation of granulomas in lymph nodes and other organs, such as the lungs and liver. Current knowledge of CL pathogenesis indicates that the induction of humoral and cellular immune responses are fundamental to disease control. The aim of this study was to evaluate the humoral and cellular immune responses in BALB/c mice inoculated with a *C. pseudotuberculosis* strain isolated in the state of Bahia, Brazil.

**Results:**

The lymphocyte proliferation and *in vitro* production of IFN-γ, IL-4, IL-10, IL-12 and nitric oxide by spleen cells stimulated with secreted and somatic antigens from the studied strain were evaluated. IgG subclasses were also analyzed. Results showed a significant increase of Th1-profile cytokines after 60 days post-inoculation, as well as an important humoral response, represented by high levels of IgG2a and IgG1 against *C. pseudotuberculosis*.

**Conclusion:**

The T1 strain of *C. pseudotuberculosis* was shown to induce humoral and cellular immune responses in BALB/c mice, but, even at a dosage of 1x10^7^ CFU, no signs of the disease were observed.

## Background

Caseous lymphadenitis (CL) is a chronic disease caused by *Corynebacterium pseudotuberculosis* that mainly affects small ruminants. Despite the economic [1, 2] and zoonotic [3] relevance of CL, a satisfactory vaccine model has not been developed [4, 5].

*C. pseudotuberculosis* is a facultative intracellular pathogen that can persist inside macrophages and stimulate the formation of granulomas [6, 7]. This species is distributed worldwide, but has the most severe economic impacts in Oceania, Africa and South America [8].

The pathogenesis of CL in mice was demonstrated by monitoring the progression of lesions in the skin and viscera of infected animals [9]. Moreover, the physiology, pathogenicity and virulence mechanisms of *C. pseudotuberculosis* strains have been elucidated using genomics [8, 10], transcriptomics and proteomics methodologies [11, 12].

The immune response against *C. pseudotuberculosis* has a well-known humoral component and involves a complex cellular mechanism against secreted and somatic bacterial antigens [13–15].

The cytokines Tumor necrosis factor-α (TNF-α) and interferon-γ (IFN-γ) are important to mount an immune response in mice as well as sheep, whether naturally infected or inoculated with *C. pseudotuberculosis* [16–19]. It is known that, with respect to *Mycobacterium tuberculosis*, a microorganism largely phylogenetically similar to *C. pseudotuberculosis*, these cytokines play a major role in susceptibility and regulation of associated lesions in mice [20].

El-Enbaawy *et al*. (2005) [21] demonstrated that antigens obtained from a *C. pseudotuberculosis* strain isolated from a naturally infected sheep, specifically a toxoid associated with bacterin, induce the production of IFN-γ, as well as elicit a humoral immune response in BALB/c mice. The present study employed a naturally attenuated strain of *C. pseudotuberculosis*, denominated T1, isolated from a granuloma taken from a goat in a rural region of the state of Bahia, located in northeastern Brazil. Studies previously conducted with this strain show that it grows quickly in BHI broth medium, when compared to other strains, but is incapable of inducing disease in goats [22–24].

The present study characterized the immune response in BALB/c mice, considering five animals per group, against antigens derived from the T1 strain of *C. pseudotuberculosis*. This murine model was chosen because of impaired IFN-γ production in response to antigens derived from *M. tuberculosis*, which is very closely related to *C. pseudotuberculosis* [25]. The proliferation of spleen cells was investigated, as well as the production of cytokines, nitric oxide (NO) and serum IgG subclasses to expand the understanding of humoral and cellular immune responses against this strain, which may represent an ideal vaccine candidate against this disease.

## Results

**Fig. 1:**
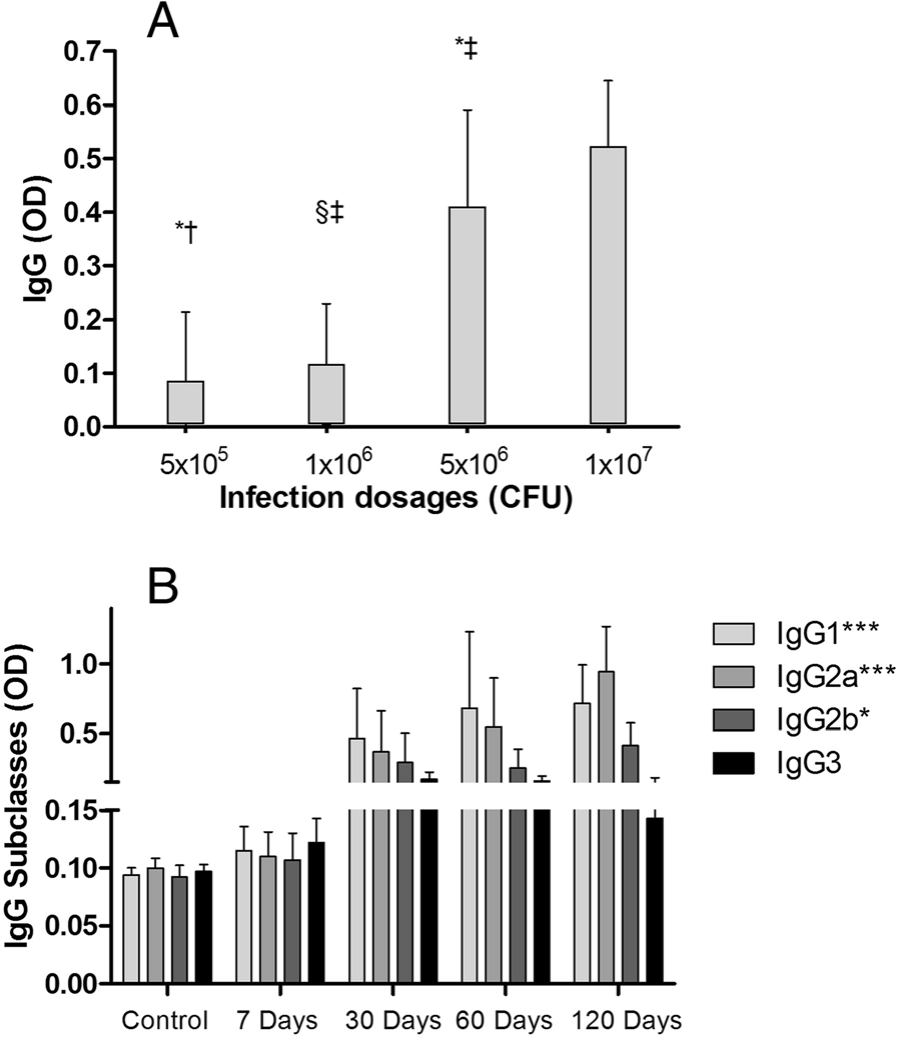
Serum IgG immune response in mice inoculated with T1 *C. pseudotuberculosis* strain, as evaluated by ELISA. Graph represents means of Optical Density (OD) values found for each group (*n* = 5 animals for group). Results are representative of the mean values obtained from two experiments. **a**. BALB/c mice were inoculated with increasing dosages: 5x10^5^, 1x10^6^, 5x10^6^ and 1x10^7^ CFU. Blood was collected 120 days after inoculation. Data were analyzed by ANOVA and Tukey post-hoc tests; *, †, ‡ and § indicate pairs with statistically significant differences. **b**. IgG subclass (IgG1, IgG2a, IgG2b and IgG3) production throughout the course of the experiment: control (before infection), 7, 30, 60, and 120 days after infection. Mice were inoculated with 10^7^ CFU of T1 strain of *C. pseudotuberculosis*. Data were analyzed by ANOVA. *P < 0.05; ***P < 0.001

To determine the optimal inoculation dosage, four different infection dosages (5x10^5^, 1x10^6^, 5x10^6^ and 1x10^7^ CFU) of the T1 *C. pseudotuberculosis* strain were tested in BALB/c mice. ELISA results showed higher IgG levels in mice infected with the two higher dosages in comparison to the two lower levels tested (*P* < 0.001) (Fig. 1a). No significant differences in IgG levels were seen between the groups inoculated with 5x10^6^ and 1x10^7^ CFU, nor in the groups inoculated with 5x10^5^ and 1x10^6^ CFU. At 120 days post-infection, none of the animals presented any evidence of lesions characteristic of the disease under clinical examination or necropsy. Because the 1x10^7^ CFU dosage was not observed to induce lesions, this experimental protocol was used to evaluate the production of IgG subclasses and cytokines.

Analysis of the humoral immune response against T1 *C. pseudotuberculosis* revealed that IgG2a production gradually increased over time, being the predominant IgG subclass at 120 days after infection (*P* < 0.001). A significant increase in IgG1 levels (*P* < 0.001) was also observed, and a discrete, yet still statistically significant, increase of IgG2b (*P* < 0.05) was seen. No statistically significant differences in IgG3 levels were detected over the course of experimentation. Control group results are representative of the mean OD readings obtained from five animals before infection (Fig. 1b).

**Fig. 2:**
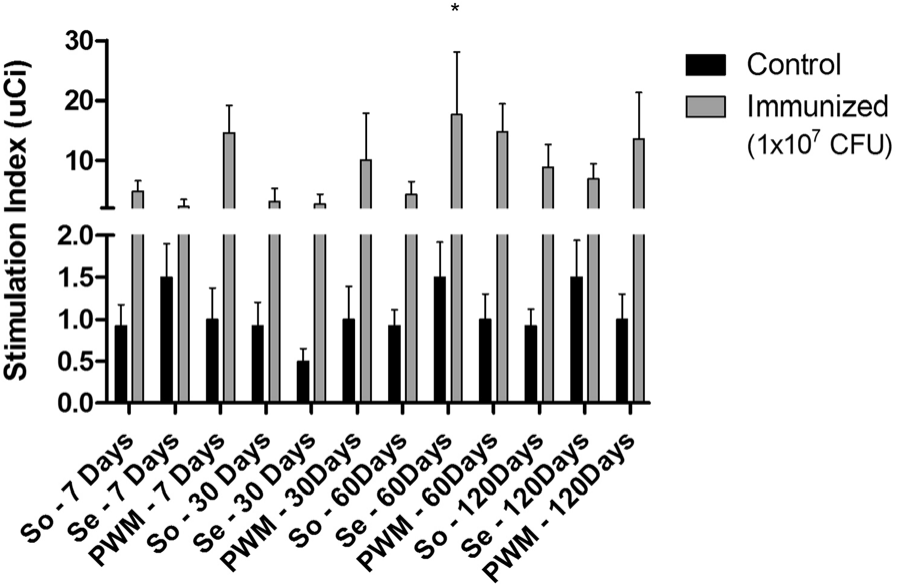
Proliferation of murine spleen cells stimulated with somatic (So) and secreted (Se) antigens. Results express the stimulation index (μCi) calculated from two independent experiments using splenocytes retrieved from five non-infected (control) and five infected (inoculated) animals from each group. Data were analyzed by ANOVA and Tukey post-hoc tests; *P < 0.05

With respect to spleen cell response to antigenic stimuli, a significant lymphoproliferative response, expressed as SI, was observed after stimulation with secreted antigen (Se) (*p* < 0.05) at 60 days post-infection in comparison to 7 and 30 days (Fig. 2). Stimulation with Se provoked a significant difference in SI in comparison to So at 60 days after inoculation.

**Fig. 3:**
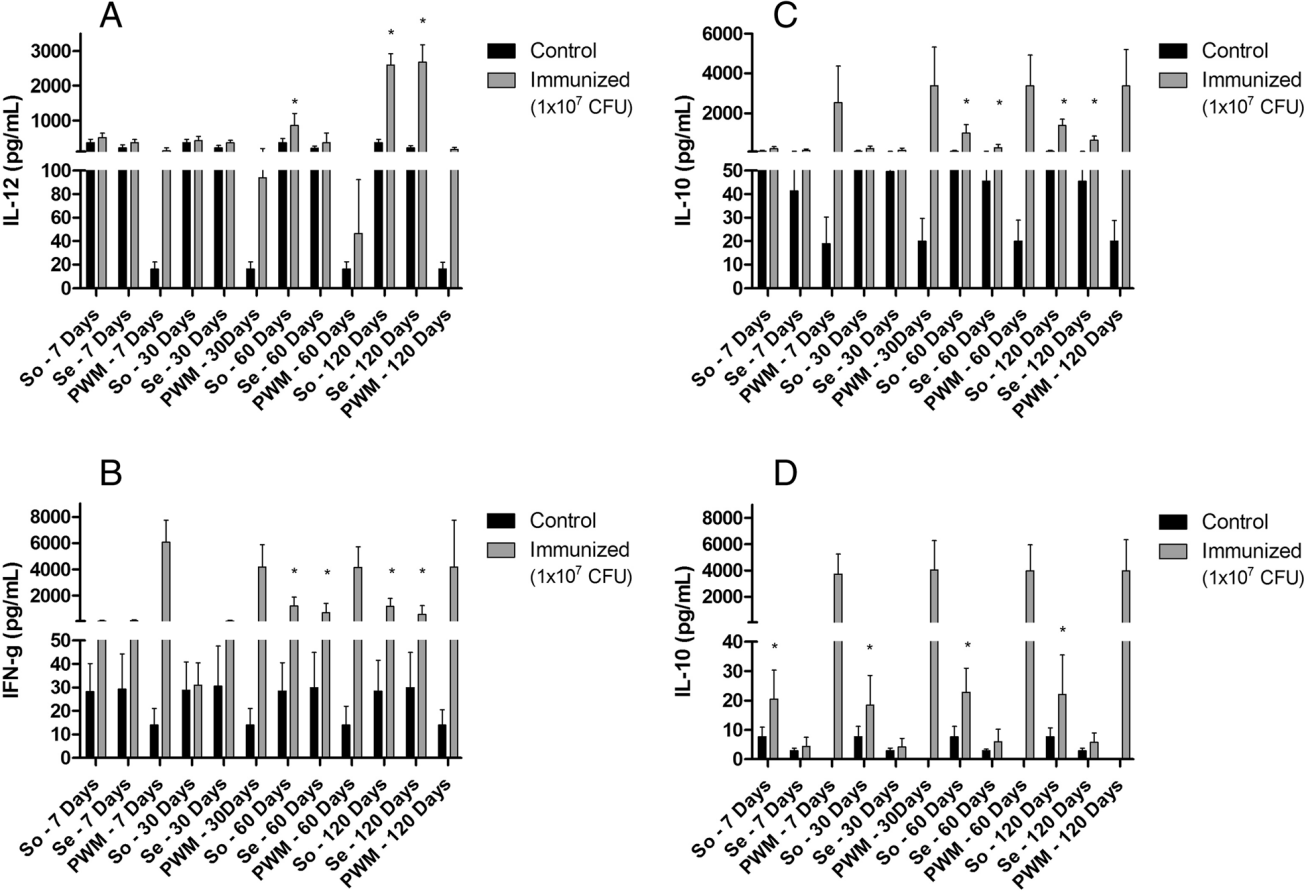
*In vitro* cytokine production by murine spleen cells stimulated with somatic (So) and secreted (Se) antigens. **a**. Interleukin-12 (IL-12). **b**. Interferon-γ (IFN-γ). **c**. Interleukin-10 (IL-10). **d**. Interleukin-4 (IL-4). Results are presented as ρg/mL, and represent the means of two independent experiments using spleen cells retrieved from five non-infected (control) and five infected (inoculated) animals from each group. Data were analyzed by ANOVA and Tukey post-hoc tests; *P < 0.05

*In vitro* production of interleukin-12 (IL-12) by spleen cells after stimulation with So or Se antigens is shown in Fig. 3a. Cell stimulated with both antigens had higher IL-12 concentrations at 60 and 120 days post-infection in comparison to controls (*p* < 0.05).

No differences were seen in IFN-γ concentration in the antigen-stimulated culture supernatants in comparison to controls at seven and 30 days post-infection, but there significant increases were observed at 60 days (p < 0.05) and 120 days (*p* < 0.05) post infection. So also induced a higher and statistical significant INF-γ production, when compared to Se stimulation (Fig. 3b) at both of these times points.

With respect to *in vitro* interleukin-10 (IL-10) production, a significant statistical difference was observed in So-stimulated cells at 60 days post-inoculation (*p* < 0,05) in comparison to the previous infection times, and also in comparison to cells stimulated with Se at this same time point. A similar situation was observed at 120 days after inoculation (Fig. 3c). In addition, cells stimulated with So induced higher levels of IL-10 than Se throughout the experiment.

Interleukin-4 (IL-4) concentrations were very low at all experiment times evaluated with respect to both antigens. However, IL-4 production by cells stimulated with So was observed to significantly increase throughout the course of investigation (*p* < 0.05), but decreased at the 120 day time point (Fig. 3d).

**Fig. 4:**
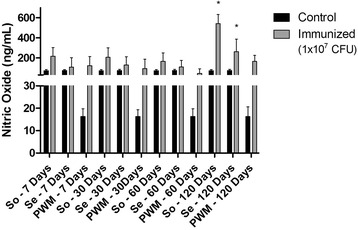
*In vitro* production of Nitric Oxide (NO) by murine spleen cells stimulated with somatic (So) and secreted (Se) antigens. Results are presented as ng/mL, and represent the means of two independent experiments using spleen cells retrieved from five non-infected (control) and five infected (inoculated) animals from each group. Data were analyzed by ANOVA and Tukey post-hoc tests; *P < 0.05

Nitric oxide (NO) production measured in the supernatant of cell cultures stimulated So and Se is illustrated in Fig. 4. A significant increase (*p* < 0.05) in NO levels was seen only at 120 days post-infection in comparison to controls.

NO production by cells stimulated with So was also observed to be higher in comparison to Se, with statistical significance (*p* < 0.05) at 120 days after inoculation.

## Discussion

The present study found that experimental inoculations of the attenuated T1 strain of *C. pseudotuberculosis* at a dosage of 10^7^ CFU did not result in lesions in BALB/c mice, even though these animals have demonstrated susceptibility to intracellular pathogens [26, 27]. Nevertheless, a previous study has shown that a wild-type strain of *C. pseudotuberculosis* was able to induce lesions at a dosage of 10^2^ [15].

In addition, cell cultures stimulated with T1 strain antigens were found to induce a proliferation of spleen cells, with secreted antigens (Se) demonstrating greater effectiveness than somatic antigens (So) two months after inoculation. A previous study showed that Se was able to enhance lymphocyte proliferation in PBMCs of an experimentally infected goat [23], which is consistent with our results. Se was found to induce a more intense proliferation than So due to the presence of phospholipase D, an exotoxin secreted by *C. pseudotuberculosis* at the beginning of infection to cleave the host cell membrane [28], which may cause a preeminent proliferation of B lymphocytes and elicit antibody production. Notably, lymphoproliferation in a murine model after stimulus with *C. pseudotuberculosis* antigens has not been described in the literature to date.

Experimental inoculation with T1 was observed to elicit high titers of IgG antibodies. The main IgG subclasses produced throughout the course of infection were IgG1 and IgG2a. As *C. pseudotuberculosis* is an intracellular pathogen that produces phospholipase D, an exotoxin with highly immunogenic properties [29, 30], the production of specific immunoglobulins is crucial to neutralize phospholipase D.

In addition, the cellular immune response is another way of reducing the dissemination of the pathogen, which can survive and multiply inside macrophages [7, 14, 18]. Accordingly, we found a significant production of IFN-γ by spleen cells after stimulation with So or Se *C. pseudotuberculosis* antigens two months after inoculation. This situation was sustained until the end of the experiment (120 days). Elevated IL-4 production was not detected, yet, in cells stimulated with So, the production of this cytokine was four times higher in comparison to those stimulated with Se and controls. This phenomenon may possibly have occurred because So has a larger amount of structural proteins and lipid antigens than Se [31]. Relatedly, Lan *et al*. (1999) [19] found a pronounced increase in IFN-γ production starting in the third week post-inoculation in splenic cell cultures of ICR-JCL mice inoculated with ATCC 1940 strain and stimulated with formalin-killed bacterial cells, which was sustained until the eighth week. In the same experiment, no significant production of IL-4 was observed.

IL-10 and IL-12 production by spleen cells stimulated by Se or So antigens increased post-inoculation time and was sustained at all time points evaluated. So was found to induce higher levels of IL-10 than Se, probably due the structural components of So, such as cytoplasm and membrane lipoproteins [31].

IL-10 may control IFN-γ synthesis during infection, thereby avoiding Th1 over-reactivity [32]. On the other hand, IL-12 can also trigger mechanisms related to cell proliferation and IFN-γ production [33]. Some studies have showed that IFN-γ, IL-10 and IL-12 are required to control persistent infections caused by intracellular parasites [34–36]. IL-12 is a cytokine crucial to Th1 shift, which is required to prevent the dissemination of pathogens within the host in order to control infection by facultative intracellular bacteria, such as *C. pseudotuberculosis* [14]. Accordingly, we found increased IL-12 production after 60 days, probably resulting from an immune response to reduce bacterial proliferation. Higher levels of IL-12 were detected at 120 days for both So and Se antigens, probably due to the persistence of infection. It is possible that, after this time, these levels would decrease as a result of IL-10 production.

NO production was also evaluated, due to its effectiveness in regulating the growth of intracellular pathogens [37]. Proteomic analysis has identified NO-responsive extracellular proteins of *C. pseudotuberculosis* and it also demonstrated the participation of the extracytoplasmic function sigma factor σ^E^ in composition of *C. pseudotuberculosis* exoproteome [38]. In the present studt, while NO production by spleen cells stimulated with So and Se antigens was higher at 120 days post-infection, So resulted in higher proliferations than Se, in accordance to what was observed in IFN-γ and IL-10 production.

## Conclusion

The attenuated T1 strain of *C. pseudotuberculosis* was found to induce both humoral and cellular immune responses in an experimental model of susceptible BALB/c mice. A 10^7^ CFU dosage did not result in any lesions in the mice evaluated. As the present study has demonstrated that, in addition to the production of antibodies, an efficient cellular response is important to the control of CL, the T1 strain can be considered as a promising option for potential vaccine candidates.

## Methods

### Bacterial strain

The T1 strain of *C. pseudotuberculosis* was isolated from granulomas obtained from goats raised in the municipality of Juazeiro, located in the state of Bahia in northeastern Brazil. Isolates were stored in the Department of Microbiology collection center at the Health Sciences Institute of the Federal University of Bahia (ICS - UFBA).

The identification of the T1 strain was confirmed by several microbiological methods: Gram staining, colony morphology, synergistic hemolytic (CAMP) reactions with *Rhodococcus equi*, urease and catalase production, as well as glucose and maltose fermentation. A commercial kit was used to aid in identification (API Coryne - BioMérieux, Merci l’Etoile, France). Since the T1 strain demonstrated a less severe pattern of hemolysis during synergistic hemolysis testing in comparison to other wild strains, other authors have suggested its use as a vaccinal strain [39].

The T1 strain was cultivated in Brain/Heart Infusion (BHI) broth and incubated for 72 h at 37 °C. The bacterial suspension was washed in Phosphate Buffer Saline (PBS) and centrifuged for 30 min at 3,000 *g* at 4 ° C.

### Somatic antigen (So)

The bacterial pellet was homogenized in PBS (pH 7.4) and sonicated at 60 Hz under 4 °C for five cycles lasting 60 s each (Branson Sonifier 450, Branson, Dunbury, CT, USA). The sample was centrifuged for 30 min at 10,000 *g* and, after collection, the supernatant was stored at -20 °C in aliquots until use. Protein concentration was determined by Lowry’s modified method using a Bio-Rad Protein Assay (Bio-Rad, Hercules, CA, USA).

### Secreted antigen (Se)

Se was obtained from the culture supernatant by saturation with 30 % ammonium sulfate (HCl) pH 4.0 and n-butanol under slow agitation at room temperature. The sample was homogenized, left undisturbed for 60 min, and then centrifuged for 10 min at 1,350 *g* under 4 ° C. The resulting interphase was dissolved in 20 mM of Tris buffer pH 7.4 (500 μL of buffer to 5 mL of culture supernatant), followed by dialysis in 50 mM Phosphate buffer pH 7.4 for 48 h. The sample was concentrated by ultra-filtration with a 10 kDa membrane (Millipore, Billerica, MA, USA). Protein concentration was determined by Lowry’s modified method using a Bio-Rad Protein Assay (Bio-Rad, Hercules, CA, USA).

### Inoculation protocol and experimental design

Prior to experimental inoculation, an optimal inoculation dose experiment was performed to obtain maximum antibody production. Eight-week-old male and female BALB/c mice, provided by the Experimental Animal Facility at the Gonçalo Moniz Research Center, Oswaldo Cruz Foundation, Salvador, Bahia-Brazil, were used to establish the inoculation protocol. The optimal dose was determined using five groups of five mice. Four groups received an intraperitoneal inoculation of 5x10^5^,10^6^, 5x10^6^ and 10^7^ colony forming units (CFU) of *C. pseudotuberculosis* T1 strain diluted in sterile PBS at a final volume of 1 mL. The control group received 1 mL of sterile PBS by intraperitoneal inoculation. Blood was collected from the tail vein and the animals used for dosage experimentation were euthanized in a CO_2_ chamber. ELISA was performed 120 days after inoculation to evaluate humoral immune response by identifying the highest levels of IgG and its subclasses.

After determining the optimal inoculation dosage, male and female BALB/c mice received intraperitoneal inoculations with 10^7^ CFU/mL of T1 strain in 1 mL of sterile PBS, while the control group was inoculated with 1 mL of sterile PBS. After blood sampling from the tail vein, five animals from each group were euthanized in a CO_2_ chamber at 7, 30, 60 and 120 days after receiving inoculation. The animals’ spleens were removed for splenocyte isolation in order to perform *in vitro* lymphocyte proliferation and cytokine production assays. Blood was also collected for immunoglobulin analysis.

### Indirect ELISA for analysis of IgG and its isotypes

ELISA plates (Costar, Corning Life Sciences, Lowell, MA, USA) were coated with So (1 μg in 100 μL of 50 mM Carbonate-bicarbonate buffer pH 9.6, in each well), incubated overnight at 4 ^o^C and washed twice in 0.05 % PBS Tween-20 (PBS-T). Plates were then blocked with 200 μL of 5 % skim milk in 0.05 % PBS-T and incubated for 2 h at 37 ^o^C. Next, the plates were washed once with PBS-T and 50 μL of diluted serum (1:50 in 1 % skim milk/PBS-T) was added to each well. Plates were then incubated for 1 h at 37 °C and washed five times with PBS-T. Next, wells were filled with 50 μL/well of HRP conjugated rabbit Ig antimouse IgG (Sigma-Aldrich, St Louis, MO, USA) at a dilution of 1:10,000 in 1 % skim milk/PBS-T to assess total IgG. To evaluate IgG1, IgG2a, IgG2b and IgG3, wells were filled with 50 μL/well of HRP conjugated rabbit Ig antimouse IgG1, IgG2a, IgG2b or IgG3 (Zymed, San Francisco, CA, USA), respectively, each diluted at 1:8.000 in 1 % skim milk/PBS-T. All plates were then incubated for 45 min at 37 ^o^C. Each plate was washed five times in PBS-T and 50 μL/well of Citrate Phosphate Buffer pH 5.1, ortho-phenyl-diamine (Sigma, St Louis, MO, USA) and 30 % H_2_O_2_] were added and left for 15 min at room temperature in a dark chamber. Reactions were stopped with 25 μL/well of 4 N H_2_SO_4_ and Optical Density (OD) was measured at 490 nm using an ELISA Plate Reader (BIORAD, Hercules, CA, USA).

### Lymphocyte proliferation assay

The spleen of each mouse was removed, washed three times with Hanks’ solution, and then placed in a petri dish containing 5 mL of RPMI 1640 medium (Gibco Laboratories, North Andover, MA, USA) supplemented with penicillin and streptomycin. The spleens were then macerated and the cellular suspension was transferred to a conical tube containing 5 mL of the same medium, followed by centrifugation at 400 *g* for 3 min. Pellets were resuspended in 0.17 M of NH_4_Cl for 5 min at 4 °C in order to lyse erythrocytes. Cells were washed 3 times with RPMI and then resuspended in RPMI enriched with 10 % bovine fetal serum.

Cell viability was determined by a Trypan Blue exclusion assay. 10^6^ cells/mL were cultivated in 96-well microculture plates in RPMI-1640 medium supplemented with L-glutamine, penicillin/streptomycin, gentamicin and 10 % fetal calf serum. Cells were stimulated by So or Se *C. pseudotuberculosis* antigens (40 μg/mL), and pokeweed mitogen (2.5 μg/mL) was used as a positive control, while medium alone was used as a negative control. All plates were incubated for 120 h at 37^o^ C under 5 % CO_2_. 1 μBq/well (10 μL) of fresh [^3^H] thymidine (GE Healthcare, Bucks, UK) was added 18 h prior to the end of the incubation time using a beta counter system (iMatic Canberra, Meriden, USA). After 120 h, plates were frozen at -20 ^o^C and β-radiation was measured as described by Paule *et al*. (2004) [23]. Results are expressed in terms of a Stimulation Index (SI), calculated by dividing the β-radiation found for each stimulated sample by the radiation measured from its respective negative control.

### ELISA for cytokines quantification

Cytokine analysis was performed in a culture supernatant obtained from cells (10^6^/mL) cultivated in the same medium used for lymphoproliferation assay. Spleen cells were stimulated with So and Se *C. pseudotuberculosis* antigens (40 μg/mL), pokeweed mitogen (2,5 μg/mL) as a positive control, and the medium alone (negative control). The plates were incubated for 120 h at 37^o^ C in 5 % CO_2_ [40]. The supernatant was collected, centrifuged, and kept at -20^o^ C until use.

Cytokine profile analysis was performed using commercial kits for IFN-γ and IL-10 (R&D Systems, Minneapolis, MN, USA), and IL-12 and IL-4 (Pharmigen, San Jose, CA, USA) according to the manufacturer instructions. Results are expressed in ρg/mL.

### Nitric oxide (NO) production assay

The presence of NO in the supernatant of spleen cells cultures that were incubated for 120 h was measured by nitrite assay, based on Griess reaction [41]. Briefly, supernatant (50 μL) was mixed with 50 μL of Griess reagent (1 % sulfanilamide and 0.1 % N-(1-naphthyl)ethylenediamine, in 5 % phosphoric acid) and incubated for 10 min at room temperature. Absorbances were measured at 492 ηm using an ELISA microplate reader (BioRad, Hercules, CA, USA). The standard curve of NO_2_^−^ was prepared by diluting nitrite stock solution (1 M NaNO_2_ diluted in Milli-Q water) in spleen cell culture media. Results are expressed in ηg/mL.

### Statistical analysis

For determination of statistical significance between experimental groups at an individual time-point, a analysis of variance (ANOVA) was performed using SPSS 12.0. (IBM Statistics, Chicago, EUA). A *p* value of <0.05 was considered significant.

## Abbreviations

CL: Caseous lymphadenitis
NO: Nitric oxide
OD: Optical density
Se: Secreted antigen
So: Somatic antigen.
